# Synthesis of Ruthenium-Promoted ZnO/SBA-15 Composites for Enhanced Photocatalytic Degradation of Methylene Blue Dye

**DOI:** 10.3390/polym15051210

**Published:** 2023-02-27

**Authors:** Dănuţa Matei, Abubakar Usman Katsina, Sonia Mihai, Diana Luciana Cursaru, Raluca Şomoghi, Cristina Lavinia Nistor

**Affiliations:** 1 Faculty of Petroleum Technology and Petrochemistry, Petroleum—Gas University of Ploiesti, 100680 Ploiești, Romania; 2 Department of Pure and Industrial Chemistry, Bayero University, Kano PMB 3011, Nigeria; 3 National Institute for Research and Development in Chemistry and Petrochemistry—ICECHIM, 060021 Bucharest, Romania

**Keywords:** mesoporous silica, ruthenium, impregnation, synthetic dyes, methylene blue, mesoscopic ordering

## Abstract

Synthetic organic pigments like xanthene and azo dyes from the direct discharge of textile effluents are considered colossal global issues and attract the concern of scholars. Photocatalysis continues to be a very valuable pollution control method for industrial wastewater. Incorporations of metal oxide catalysts such as zinc oxide (ZnO) on mesoporous Santa Barbara Armophous-15 (SBA-15) support to improve catalyst thermo-mechanical stability have been comprehensively reported. However, charge separation efficiency and light absorption of ZnO/SBA-15 continue to be limiting its photocatalytic activity. Herein, we report a successful preparation of Ruthenium-induced ZnO/SBA-15 composite via conventional incipient wetness impregnation technique with the aim of boosting the photocatalytic activity of the incorporated ZnO. Physicochemical properties of the SBA-15 support, ZnO/SBA-15, and Ru-ZnO/SBA-15 composites were characterized by X-ray diffraction (XRD), N_2_ physisorption isotherms at 77 K, Fourier-transform infrared (FTIR), scanning electron microscopy (SEM), energy dispersive X-ray (EDS), and transmission electron microscopy (TEM). The characterization outcomes exhibited that ZnO and ruthenium species have been successfully embedded into SBA-15 support, andtheSBA-15 support maintains its structured hexagonal mesoscopic ordering in both ZnO/SBA-15 and Ru-ZnO/SBA-15 composites. The photocatalytic activity of the composite was assessed through photo-assisted mineralization of aqueous MB solution, and the process was optimized for initial dye concentration and catalyst dosage. 50 mg catalyst exhibited significant degradation efficiency of 97.96% after 120 min, surpassing the efficiencies of 77% and 81% displayed by 10 and 30 mg of the as-synthesized catalyst. The photodegradation rate was found to decrease with an increase in the initial dye concentration. The superior photocatalytic activity of Ru-ZnO/SBA-15 over the binary ZnO/SBA-15 may be attributed to the slower recombination rate of photogenerated charges on the ZnO surface with the addition of ruthenium.

## 1. Introduction

Over the past few decades, water contamination, as a result of increased industrial activities, has adversely affected human health and the quality of the aquatic ecosystem [1]. The major lethal contaminants present in water and wastewater include synthetic organic dyes [2,3], fluorides [4], uranium [5], heavy metals [6], and pharmaceutically active compounds (PhACs) [7]. Synthetic organic dyes are hazardous, non-biodegradable materials that massively contribute to water pollution. They have been linked to adverse health effects that include dermatitis [8], cancer [9], organ dysfunctions [10], and birth defects [11]. These substances are used in a wide variety of products, including clothing, textiles, and food packaging [12]. While they are not toxic in and of themselves, synthetic organic dyes can become toxic when they leach into the environment or when they accumulate in people’s bodies [13]. Methylene blue (MB) is among such recalcitrant synthetic organic dye pollutants, and it is found in many industrial sources and their manufacturing facilities. These include its use as a coloring agent for paper, plastics, and textile industries [14,15], food coloring for the agricultural industry [16], bacteria identification agent, anti-aging compound, and antioxidant in pharmaceutical and cosmetics industries [17,18]. The dye has been found to cause harmful effects on humans, animals, and fish. The most serious health effects associated with methylene blue are eye and skin irritations, nausea, headaches, diarrhea, headache, mutations, and abdominal pain [19]. Since some of these highly stable organic dyes cannot be influenced by temperature or light, their elimination from water effluents has become an important area of research. As a result, removing methylene blue and other organic dyes from water is crucial if we want to protect ourselves from their toxic effects, and our aquatic ecosystems survive into the future.

Green technologies like adsorption [20] and advanced oxidation processes (AOPs) [21,22], along with conventional wastewater treatment methods (WWTMs) such as coagulation [23], chemical oxidation [24], membrane filtration [25], and bio-process [26,27], remain the most frequent WWTMs employed for the removal of MB and other synthetic organic dyes. All but AOPs have tendencies to produce secondary contaminants as by-products, and are reported to be ineffective in the complete removal of harmful dyes [28]. AOPs like heterogeneous photo-induced catalysis, on the other hand, are reported to be potent in the removal of dyes and other contaminants with only carbon dioxide and water as the products of the reaction [29]. Additionally, adsorption and photocatalytic degradation of dyes and nitro-compounds are viewed as the most economical and proficient water pollution control technologies [30]. Recently, Usman et al. recently employed anatase phase {001}-TiO_2_/Au hybrid nanocomposites for successful photocatalytic removal of aqueous MB dye [31]. Similarly, Sidra et al. [32] reported the development of a cost-efficient rGO-Fe_3_O_4_/TiO_2_photocatalyst, and successfully employed it for photocatalytic degradation of MB and malachite green under UV-visible light illumination. In the same vein, Obey et al. [33] employed a biochar derived from a non-customized matamba fruit shell for efficient wastewater treatment through adsorption.

Over the past few decades, well-organized mesoporous silica sieves, metal-organic frameworks, and zeolites were employed as support materials for catalysts among other applications, owing to their large surface area and pore volume [34]. Well-ordered mesoporous silica sieves exhibit high specific surface area and pore volumes of up to 1500 m^2^·g^−1^ and 1 cm^3^·g^−1^, respectively. An example of ordered mesoporous silica sieves is Santa Barbara Amorphous-15 (SBA-15). SBA-15 is a thermally and mechanically stable, inert, as well as eco-friendly material with a 2D hexagonal pore structure [35]. These properties, along with its high surface area (400–900 m^2^·g^−1^) and a uniform pore size distribution make it effective in addressing the aggregate formation issue of metal oxide catalysts [36]. Characteristically, no significant diffraction peak can be observed in wide-angle XRD diffraction analysis because the silica on the SBA-15 structure is generally amorphous [37]. Various catalytic materials such as metal oxides, precious metals, and non-noble metal sulfides can be supported by SBA-15. The active specie can be dispersed in its large surface area which results in increasing the surface area of the active phase, thereby increasing its photocatalytic activity [38].

Metal-oxide-based catalysts such as ZnO and TiO_2_ are the most common photocatalytic materials reported owing to their strong light absorption performance in the UV region. These catalysts can also be used to modify the internal and external surface of mesoporous silica supports for efficient pollutants degradation. However, photocorrosion, optical absorption, agglomerates formation, and short lifetime of photoexcited electrons and holes remain the critical issues associated with ZnO-based photocatalysts [39,40]. To address these problems and enhance their photocatalytic performance, many works have been reported to employ various approaches. For example, fine-tuning the structural morphology and physicochemical properties of ZnO with mesoporous silica greatly enhances its surface area, light absorption performance, and stability of electron-hole pairs. Additionally, the wide bandgap of ZnO can be reduced through either doping with metals such as Ru [41], Au [42], Ag [43], and Pt [44], or through coupling it with other semiconductor catalysts [45,46,47].

Proficient construction of heterojunction systems is considered among the best ways of improving the activity of semiconductor-based catalysts for photocatalytic applications. A heterojunction system is a junction interface formed by the hybridization of two dissimilar semiconductors or metals [48]. Through appropriate interface formation in heterojunction systems, visible light utilization can be enhanced to a wider wavelength region, and separation efficiency can be improved [49]. Similarly, the integration of another photocatalytic material can bring about an improvement in the photocatalytic activity [50]. Through this, ZnO has been successfully combined with other materials to improve photocatalytic activity by slowing down the recombination rate of photoexcited charge carriers and widening the visible light response range. Recently, silver-decorated ZnO was reported by the combination of ZnO and Ag nanoparticles (NPs) [51]. The coupling was successfully used for solar-assisted degradation of red azo dye and ofloxacin antibiotic. Enhanced photocatalytic activity of the Ag-modified ZnO was obtained due to broadened light absorption range and improved anti-photo corrosion property. Ru-doped ZnO for photocatalytic degradation of 4-chlorophenol in water was also reported [52]. Photodegradation of 4-chlorophenol was favored because the combined rate of the photogenerated electron-hole pairs was restricted through the charge transfer between Ru^6+^/Ru^4+^ and ZnO NPs along with the formation of free radical oxidant species.

Furthermore, a combination of ZnO and SBA-15 has been extensively reported to have high photocatalytic activity under UV irradiation for the degradation of dyes in wastewater. Improved photocatalytic activity can be attributed to the improved stability provided by the support material. Recently, Metal oxides semiconductors of ZnO, TiO_2,_ and SnO_2_ deposited on a highly ordered mesoporous SBA-15 support were compared for the first time as photocatalysts in the elimination of aqueous methylene blue (MB) dye from neutral aqueous solutions by adsorption and photodegradation under UV illumination [53]. All SBA-15-supported photocatalysts exhibited an excellent capacity for aqueous MB adsorption, especially the ZnO/SBA-15 catalyst, with 2–5 times higher photocatalytic activity than the corresponding commercial ZnO, TiO_2_, and SnO_2_ nanopowders. This was attributed to the higher specific surface area provided by the SBA-15 support to the catalysts, which resulted in their higher adsorption capacity and better dispersion of the supported metal oxides.

In this present work, we synthesized Ru-promoted ZnO/SBA-15 composites via a facile impregnation technique, with the aim of improving its charge separation efficiency and subsequently assessing its photo-assisted catalytic response towards MB dye removal from aqueous solution. While there are many works reported on ZnO incorporation on SBA-15 support for organic dye removal from industrial effluents, the construction of ZnO/SBA-15 composite modified by ruthenium for photocatalytic degradation of dyes is novel and has not been to our knowledge explored previously.

## 2. Materials and Methods

The following chemicals were used in the synthesis of our catalysts: Ethanol (96%, Chemical Ch-C, Iași, Romania); Polyethylene glycol (Sigma Aldrich, Darmstadt, Germany); Pluronic P_123_ triblock copolymer (EO_20_PO_70_EO_20_, 5800, Sigma Aldrich) and Tetra-Ethyl Ortho-Silicate (TEOS, 98%, Sigma Aldrich) as an organic template and silica sources; Zinc acetate dihydrate (99.5%, Merck, Darmstadt, Germany) and ruthenium chloride (55%, Sigma Aldrich) as zinc and ruthenium precursors. All reagents were used directly without further treatment.

### 2.1. Synthesis of SBA-15 Support

Mesoporous SBA-15 was prepared using the synthesis route reported by Zhao [54] with minor modifications. Briefly, 6 g triblock copolymer, P_123_ was dissolved into a mixture of deionized water and 35% HCl. A transparent solution was obtained after 6 h of continuous mechanical stirring of the solution at 308 K. Subsequently, 12.49 g of TEOS was slowly added into the aforementioned solution to obtain a gel-like mixture. The mixture was then transferred into a Teflon bottle for aging at 373 K for 24 h. After natural cooling, the product was filtered, washed severally, and dried at 333 K. To obtain the mesoporous SBA-15 powder, the as-prepared SBA-15 was then finally calcined at 823 K in the air to get rid of the organic template.

### 2.2. Synthesis of Ru-ZnO/SBA-15 Composites

ZnO/SBA-15 composites were first synthesized via the impregnation method reported by Quach Nguyen et al. [55]. First, zinc acetate solution was prepared by dissolving 0.903 g zinc acetate in 42 mL ethanol with stirring at 343 K for 120 min. The resulting solution was allowed to naturally cool down and used for impregnation with as-prepared SBA-15. In a typical procedure, 0.5 g of SBA-15 was dispersed in the zinc acetate solution under vigorous stirring. The mixture was then oven-dried at 353 K for 24 h. The sample obtained was calcinated in the air by increasing the room temperature up to 823 K for 60 min, and then holding it at the same temperature for another 60 min. In the preparation of Ru-ZnO/SBA-15, the incipient wetness impregnation method by Okal et al. was adapted with minor modifications [56]. 1.0 g of the as-synthesized ZnO/SBA-15 was dissolved in a Ru-precursor solution containing RuCl_3_ and ethanol under continuous, room-temperature stirring for 4 h. The obtained mixture was oven-dried at 373 K for 48 h.

### 2.3. Characterization

#### 2.3.1. Textural Analysis

The textural properties of the as-synthesized SBA-15 support, ZnO/SBA-15, and Ru-ZnO/SBA-15 composites were analyzed by N_2_ physisorption isotherms under 77 K using Quantachrome Nova 2200e (BET surface area, pore volume, and pore size distribution analyzer; Quantachrome Instruments, Boynton Beach, FL, USA). The samples were vacuum-degassed at 250 °C for 4 h prior to the analysis. The properties were calculated using the NovaWin software (Boynton Beach, FL, USA).

#### 2.3.2. Structural Characterization

The structures of SBA-15 support, ZnO/SBA-15, and Ru-ZnO/SBA-15 composites were investigated by Fourier-transform infrared (FT-IR). FT-IR spectra (4000–400 cm^−1^) were measured in the Shimadzu IRTracer-100 FT-IR spectrophotometer (Kyoto, Japan). Small-angle X-ray diffraction (SA-XRD) and wide-angle X-ray diffraction (WA-XRD) modes were implemented for the structural analysis of the support and composites using a Bruker D8 Advance diffractometer (Karlsruhe, Germany; θ-θ type) with characteristic CuKα radiation (λ = 1.5418 nm) and graphite monochromator operated at 40 kV and 40 mA. X-ray diffraction (XRD) pattern for WAXS was measured in the 10 and 70° (2θ) range and scan speed of 0.1°/5 s, whereas the XRD pattern for SAXS was recorded in 2θ measurement range between 0 and 10° and a scan speed of 0.1°/5 s. Diffracplus Basic software and the PDF-ICDD 2-2008 database were used for phase identification, while quantitative analysis was carried out with the Diffracplus TOPAS 4.1 software (Karlsruhe, Germany). FT-IR spectra (4000–400 cm^−1^) were recorded as KBr pellets in a Shimadzu IR affinity-1 spectrophotometer.

#### 2.3.3. Morphology and Elemental Composition Analysis

Structural morphology images of the SBA-15 support, as well as the ZnO/SBA-15 and Ru-ZnO/SBA-15 composites, were obtained using a Scios 2 HIVAC Dual-Beam ultra-high-resolution FIB-SEM (ThermoFisher, Brno, Czech Republic). The elemental composition analysis was performed with EDAX energy dispersive X-ray (EDX) detector mounted on the same equipment. To further examine the structural and chemical nature of the as-prepared materials, high-resolution transmission electron microscopy (TEM) was performed using an FEI Tecnai G2 F-20 TWINCryo-TEM (FEI American Company, Brno, Czech Republic) operated at an acceleration voltage of 200 kV with the magnification of 80,000 and 20,000.

### 2.4. Photocatalytic Study

The photocatalytic activity of the as-synthesized heterostructured composites was investigated for degradation of aqueous MB solution using a Toption photochemical reactor, (TOPTION INSTRUMENT Co., Ltd., Xi’an China). For optimization, different masses (10 mg, 30 mg, and 50 mg) of the Ru-induced ZnO/SBA-15 catalysts, and the initial concentration of the dye pollutant were investigated at pH = 6.7. In a typical experiment, an appropriate mass of the Ru-induced ZnO/SBA-15 catalysts were dispersed in a 50 mL aqueous MB (30 mg/L) solution with stirring and then illuminated with the photoreactor. For comparison, ZnO/SBA-15 composite was also evaluated in a similar procedure. The irradiation process was monitored over 120 min for all photodegradation experiments. An appropriate portion of the contaminated solution was respectively collected at 15- and 30-min intervals for (10 mg)-Ru-ZnO/SBA-15 and (50 mg)-Ru-ZnO/SBA-15 to observe the MB removal using a Shimadzu 3600iPlus UV–Vis spectrophotometer.

## 3. Results

### 3.1. Characterization of the Materials

#### 3.1.1. Morphology and Elemental Microanalysis

**Figure 1:**
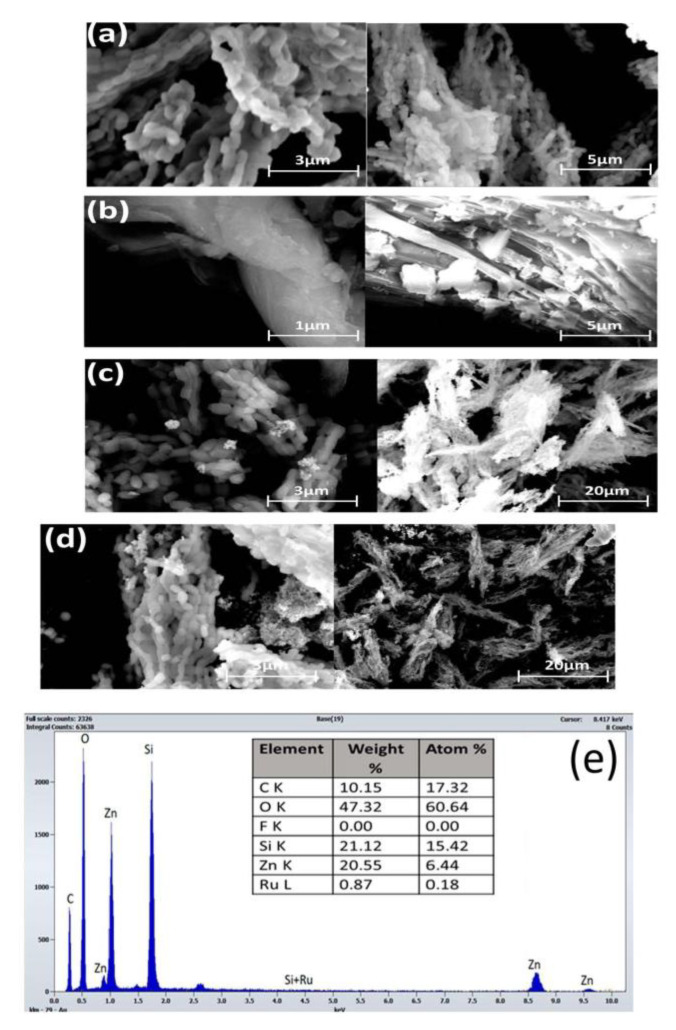
SEM images ofthe as-prepared (**a**) SBA-15; (**b**) ZnO; (**c**) ZnO/SBA-15;(**d**) Ru-ZnO/SBA-15; (**e**) and EDX of Ru-ZnO/SBA-15.

Structural morphologies of the as-prepared samples were analyzed with SEM as displayed in Figure 1 below. Figure 1a shows a packed agglomerate of rope-shaped morphology with an average size of 0.57 µm. This is consistent with the well-known 2D hexagonal SBA-15 structure [57]. Although small debris of the introduced species was observed on the amorphous silica surface in Figure 1c,d, the diminished SA-XRD peaks intensity from XRD data (Section 3.1.4), and the amount of adsorbed nitrogen in N_2_physisortion data (Section 3.1.3) suggest that ZnO and Ru particles are embedded into the SBA-15 mesopores. The SEM images in Figure 1c,d, exhibit a well-organized two-dimensional hexagonal structure, which confirmed that the mesoscopic ordering of the amorphous SBA-15 is maintained with the incorporation of both ZnO and Ruthenium species. The EDX spectra of the Ru-ZnO/SBA-15 composite in Figure 1e reveal that our samples contain Si, Zn, O, Ru, and C as constituent components. The trace of carbon is due to the sample coating in SEM imaging. The high-intensity peaks observed for Si, Zn, and Oprove that the sample contains mainly SBA-15 and ZnO with small traces of metallic ruthenium and ruthenium oxide.

**Figure 2:**
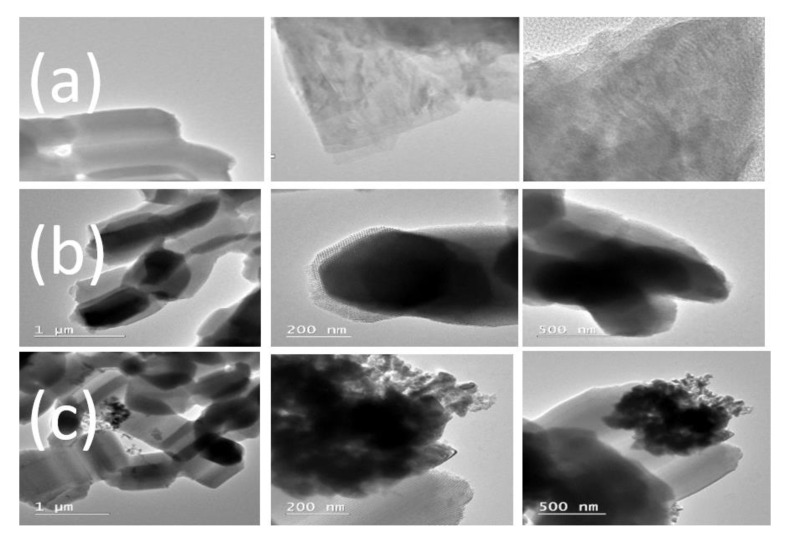
TEM images of the as-prepared (**a**) SBA-15; (**b**) ZnO/SBA-15; and (**c**) Ru-ZnO/SBA-15.

Figure 2 shows the TEM images of the as-synthesized samples of SBA-15, ZnO/SBA-15, and Ru-ZnO/SBA-15 composites. The images clearly show that the incorporation of ZnO in Figure 2b and Ru in Figure 2c has not entirely changed the regular hexagonal well-organized mesostructures of the SBA-15 support. No significant formation of ZnO or ruthenium species was seen on the external surface of the SBA-15 support. However, small debris of agglomerated particles could be observed in Figure 2c. This suggests that the impregnated ZnO and ruthenium species have been well-dispersed within the inner surface walls of SBA-15 in agreement with the N_2_ sorption data and XRD measurements.

#### 3.1.2. FTIR Measurements

**Figure 3:**
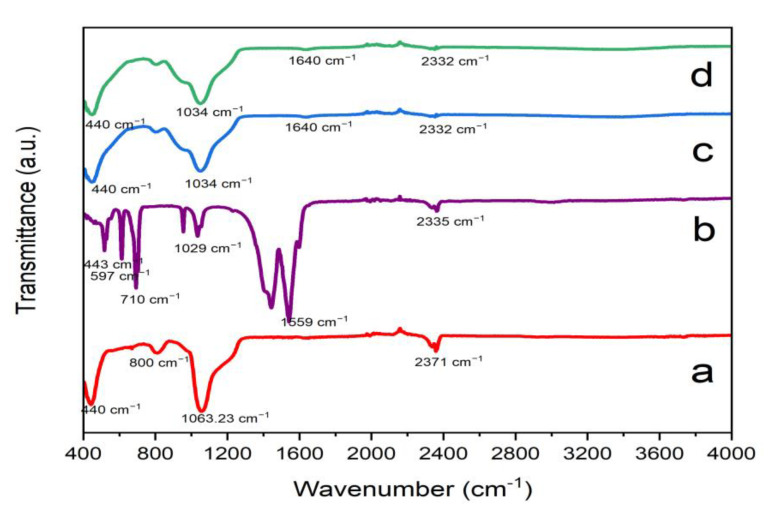
FTIR spectra of the as-prepared (**a**) SBA-15 supports; (**b**) ZnO; (**c**) ZnO/SBA-15; and (**d**) Ru-ZnO/SBA-15 composites.

The FT-IR spectra of the as-prepared SBA-15 support, ZnO/SBA-15, and Ru-ZnO/SBA-15 composites in wavenumber range from 400 to 4000 cm^−1^ are displayed in Figure 3 below. The peaks around 1063 cm^−1^, 800 cm^−1^and 400 to 467 cm^−1^ in Figure 3a correspond to distinctive Si-O and Si-OH tensile vibrations that can be found in the configuration of silica ordering network [58]. Peak associated with O=C=O compensation and H-O-H vibration of a cluster of water of crystallization is observed at 2371 cm^−1^, which intensified in both the spectra of ZnO/SBA-15 and Ru- ZnO/SBA-15 composites. The peaks at 443 cm^-1^ and 1029 cm^−1^ in Figure 3b correspond to Zn-O stretching [59]. All the peaks that correspond to SBA-15 are also observed in ZnO@SBA-15 and Ru-ZnO@SBA-15 composites, with a little shift of 1063 cm^−1^peak in Figure 3a to 1034 cm^−1^ in both Figure 3c,d, which suggests that the impregnation of ZnO has on SBA-15 has occurred. However, all the peaks except the one recorded at 2371 cm^−1^, appeared with reduced intensities due to the presence of ZnO materials.

#### 3.1.3. Sorption Measurements

**Figure 4:**
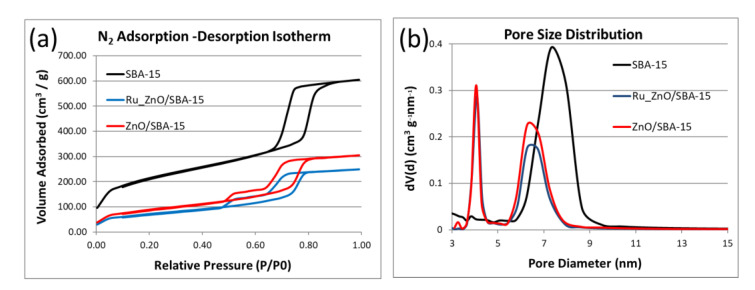
Textural analysis of the as-prepared SBA-15 supports: ZnO/SBA-15 and Ru-ZnO/SBA-15 composites: (**a**) N_2_ adsorption-desorption isotherms; (**b**) BJH pore size distribution.

The surface and pore structure analysis of the SBA-15 support, ZnO/SBA-15, and Ru-ZnO/SBA-15 composites were performed using N_2_physisortion. Figure 4a shows that all the as-synthesized materials exhibit 2015 IUPAC-classified type IV isotherms [60]. The SBA-15 support demonstrates an H1-type hysteresis loop, a distinctive attribute of a narrow range, cylindrical homogeneous mesoporous materials [61], while ZnO/SBA-15 and Ru-ZnO/SBA-15 composites reveal atypical H5-type hysteresis loop, precisely corresponding to partly blocked mesoporous structure [62]. The observed decrease in BET surface area (S_BET_), pore diameter(D_p_), and pore volume (V_p_) in Table 1 implies the moderate plugging and covering of the pore sites of the SBA-15 supports from the introduction of both ZnO and ruthenium species. However, their incorporation did not alter with the mesoporous network of the SBA-15 supports. Figure 4b illustrates a unimodal dispersed BJH pore size distribution (PSD) curve (7.3 nm) for SBA-15 support and bimodal PSD curves (4.1 and 6.3 nm) for ZnO/SBA-15 and Ru-ZnO/SBA-15 composites.

#### 3.1.4. X-ray Diffraction

**Figure 5:**
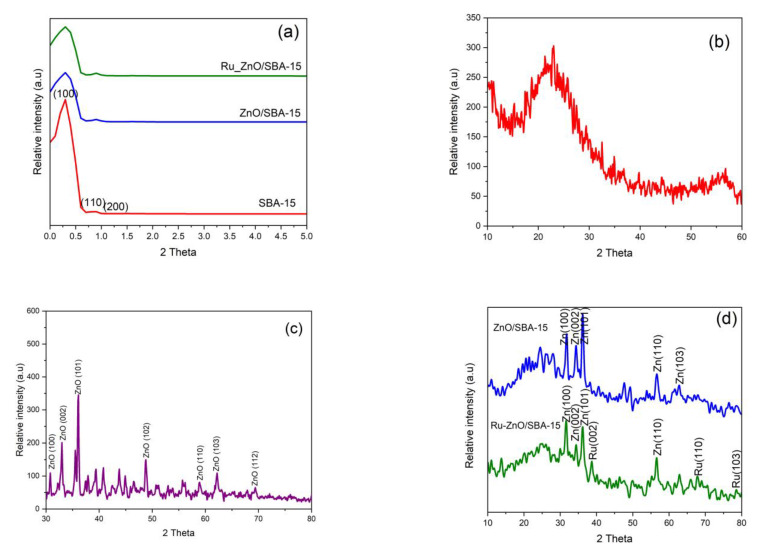
SA-XRD patterns for (**a**) as-prepared amorphous SBA-15 support, ZnO/SBA-15 and Ru-ZnO/SBA-15 composites; WA-XRD patterns for (**b**) SBA-15; (**c**) ZnO; (**d**) ZnO/SBA-15 and Ru-ZnO/SBA-15 composites.

Figure 5 below displays the SA-XRD and WA-XRD patterns of as-synthesized SBA-15 support, ZnO/SBA-15, and Ru-ZnO/SBA-15 composites. The small/low angle XRD patterns from Figure 5a reveals highly intense and mild diffractograms at 0.35° and 0.9° (2θ) corresponding to the (100) and (200) Bragg’s reflection planes respectively. This precisely depicts the hexagonal symmetry and elongated mesoporous arrangement of amorphous SBA-15 support [63]. The preservation of these reflection planes in both ZnO/SBA-15 and Ru-ZnO/SBA-15 composites shows that the incorporation of the ZnO and ruthenium species did not practically change the mesoscopic ordering of the SBA-15, and that the species were homogeneously dispersed. However, the observed decline in the intensity of aforementioned reflection planes in ZnO/SBA-15 and Ru-ZnO/SBA-15 composites, suggests the agglomeration and partial blocking of the SBA-15 pores illustrated in N_2_ adsorption-desorption isotherms results in Section 3.1.3 above.

WA-XRD patterns in Figure 5b reveal a distinctive broad band of amorphous SBA-15 between 15 and 34° (2θ) values in all the samples. Figure 5c shows the XRD diffraction patterns at 31.91, 34.52, 36.47, 47.82, 56.91, 62.74, and 68.21, corresponding to (100), (002), (101), (102), (110), (103), and (112) ZnO crystal reflection planes respectively [64]. In the case of the Ru-ZnO/SBA-15 composite (Figure 5d, six 2θ measurements at 31.9, 34.4, 36.4, 47.8, 56.6, and 62.8° that correspond to (100), (002), (101), (102), (110) and (103) reflection planes were still recorded besides the broad band of SBA-15. These diffractograms evidently match up the hexagonal structure of pristine ZnO [65]. Similarly, in addition to the broad characteristic band of amorphous SBA-15 and ZnO reflection planes, a less intense peak at 2θ = 69° that respectively matches up the (110) miller indices of the metallic form of face-centered-cubic (FCC) ruthenium [66]. These results confirm the successful incorporation of ruthenium and ZnO in the porous network of the highly disordered (amorphous) SBA-15 material.

#### 3.1.5. Photodegradation of MB

**Figure 6:**
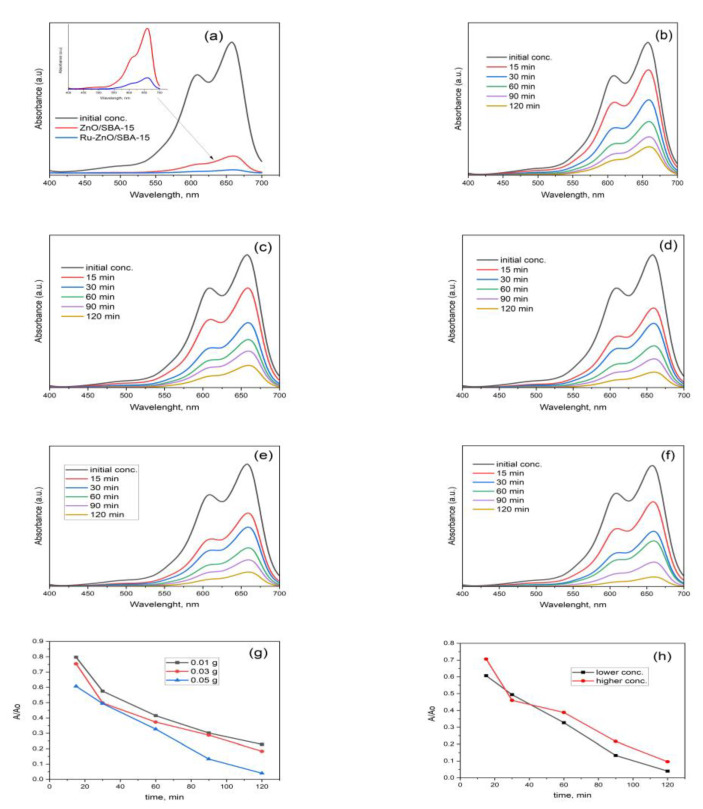
Absorbance data of MB removal for: Comparison between ZnO/SBA-15 and Ru-ZnO/SBA-15 composites (**a**); Ru-ZnO/SBA-15 at 0.01 g (**b**); 0.03 g (**c**); 0.05 g (**d**); initial MB conc. of 20 ppm (**e**) 35 ppm (**f**); photocatalytic degradation of MB in the presence of catalyst dosage (**g**); initial concentration of the pollutant (**h**).

The photocatalytic activities of the as-prepared materials were evaluated by the removal of the aqueous MB solution. Figure 6b shows the absorbance data of methylene blue removal for 50 mg of the prepared Ru-ZnO/SBA composites. It can be observed that the absorption of methylene blue decreases quickly within the first 30 mins, and the degradation efficiency reaches about 98% in 120 min irradiation. However, for the 10 and 30 mgRu-ZnO/SBA-15 catalyst, the absorption of MB (Figure 6g) decreases slower in relation to the 50 mgRu-ZnO/SBA-15. Degradation efficiencies of about 77 and 81% were recorded for 10 mg Ru-ZnO/SBA-15 and 81% for 30 mg Ru-ZnO/SBA-15 after 120 min. This was performed to establish the appropriate dose of the catalyst needed for the degradation experiment. The observed increase in the photodegradation rate from 10 mg to 50 mg diminished when the catalyst dose reached 60 mg, indicating aggregate formation that inhibits efficient light absorption. For comparison, the photodegradation of MB for ZnO-SBA-15 composite under the same conditions for 120 min is also shown in Figure 6a. A superior catalytic activity is obtained for the Ru-induced catalyst over ZnO/SBA-15 composite without ruthenium. Two concentrations of MB solution (20 ppm and 35 ppm) were used to investigate the photodegradation rate and results are displayed in Figure 6f,h. With the increase in MB concentration, the rate of photodegradation decreased. In the current work, a 20 ppm solution of MB exhibited 98% photodegradation efficiency, whereas increasing the concentration to 35 ppm showed reduced degradation efficiency at the same conditions.

**Figure 7:**
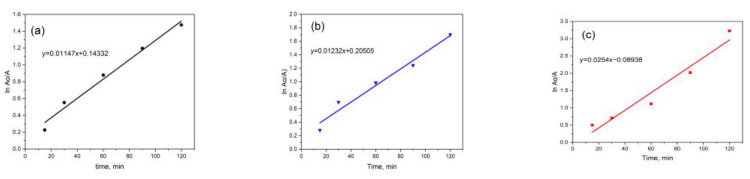
Pseudo-first order kinetics of MB degradation over different catalyst dosages of Ru-ZnO/SBA-15: (**a**) 10 mg; (**b**) 30 mg; and (**c**) 50 mg.

Figure 7 shows the photocatalytic effect of the Ru-ZnO/SBA-15 composites on the degradation rate of MB. The rate can be ascribed to the pseudo-first-order. Applicability of pseudo-first-order kinetics with apparent rate constants of 0.01147 (Figure 7a), 0.01232 (Figure 7b), and 0.02540 (Figure 7c) for MB were recorded.

## 4. Conclusions

The Ru-promoted surface of the ZnO/SBA-15 composite was successfully prepared via the conventional incipient wetness impregnation technique. Characterization results showed that the incorporated ZnO and ruthenium species were embedded into SBA-15 support without changing its mesoscopic ordering. The as-prepared Ru-ZnO/SBA-15 composite was found to have a superior photocatalytic activity over both the SBA-15 and binary ZnO/SBA-15 composite. Increased recombination time of photoexcited electron-hole pairs on the ZnO/SBA-15 surface with the addition of metallic ruthenium was understood to be responsible for its excellent catalytic activity. This work will help develop strategies for future photocatalytic degradation of other contaminants present in water and wastewater. Moreover, we are looking forward to using this photocatalytic heterojunction system for the degradation processes of PhACs. 

## Figures and Tables

**Table 1:** Physicochemical properties of the as-synthesized SBA-15 supports: ZnO/SBA-15 and Ru-ZnO/SBA-15 composites.

Catalyst	S_BET_ (m^2^/g)	V_T_ (cm^3^/g)	V_P_ (cm^3^/g)	D_P_ (nm)	S_T_ (m^2^/g)	V_M_ (cm^3^/g)
SBA-15	734	0.3941	0.766	7.3	8.3	0.015
ZnO/SBA-15	313	0.4726	0.472	4.1	-	-
Ru-ZnO/SBA-15	252	0.3852	0.388	4.1	-	-

## Data Availability

Not applicable.
